# Evaluation of Zuo-Gui Yin Decoction Effects on Six CYP450 Enzymes in Rats Using a Cocktail Method by UPLC-MS/MS

**DOI:** 10.1155/2022/4293062

**Published:** 2022-08-26

**Authors:** Bangzhen Hong, Shizhong Hong, Xuerui Hu, Fan He, Xiaoxiao Shan, Lei Wang, Weidong Chen

**Affiliations:** ^1^School of Pharmacy, Anhui University of Chinese Medicine, Hefei, Anhui, China; ^2^Institute of Drug Metabolism, Anhui University of Chinese Medicine, Hefei, Anhui, China; ^3^School of Medical Economics and Management, Anhui University of Chinese Medicine, Hefei, Anhui, China; ^4^Anhui Province Key Laboratory of Chinese Medicinal Formula, Hefei, Anhui, China; ^5^Synergetic Innovation Center of Anhui Authentic Chinese Medicine Quality Improvement, Hefei, Anhui, China

## Abstract

**Background:**

Zuo-Gui Yin Decoction (ZGYD), a traditional Chinese prescription, is mainly used in various kinds of andrology and gynecology diseases. However, the study on the interaction of ZGYD and drugs has not been reported. Therefore, evaluating the interaction between ZGYD and metabolic enzymes is helpful to guide rational drug use.

**Objective:**

This study was conducted to explore the effects of ZGYD on the activity and mRNA expressions of six Cytochrome P450 (CYP450) enzymes in rats and to provide a basis for its rational clinical use.

**Methods:**

Sprague-Dawley rats were randomly divided into control, ZGYD high, medium, and low-dose group (*n* = 6). The concentrations of six probe substrates in plasma of rats in each group were determined by UPLC-MS/MS. In addition, RT-PCR and Western blot were used to determine the effects of ZGYD on the expression of CYP450 isoforms in the liver.

**Results:**

Compared with the control group, the main pharmacokinetic parameters AUC_(0-t)_, AUC _(0~∞)_, of omeprazole, dextromethorphan, and midazolam in the high-dose group were significantly decreased, while the CL of these were significantly increased. The gene expressions of CYP2C11 and CYP3A1 were upregulated in the ZGYD medium, high-dose group. The protein expression of CYP2C11 was upregulated in the high-dose group, and the protein expression of CYP3A1 was upregulated in the medium, high-dose group.

**Conclusion:**

The results showed that ZGYD exhibited the induction effects on CYP2C11 and CYP3A1 (CYP2C19 and CYP3A4 in humans) in rats. However, no significant change in CYP1A2, CYP2B1, CYP2C7, and CYP2D2 activities was observed. It would be useful for the safe and effective usage of ZGYD in clinic.

## 1. Introduction

Zuo-Gui Yin Decoction (ZGYD), a traditional Chinese prescription, is included in “Jing Yue Quanshu” written by Jiebin Zhang of the Ming Dynasty. It is a famous prescription for treating kidney Yin deficiency [1, 2], which composes of *Rehmannia glutinosa* Libosch., *Dioscorea opposite* Thunb., *Lycium barbarum* L., *Glycyrrhiza uralensis* Fisch., *Poria cocos* (Schw.) Wolf, and *Cornus officinalis* Sieb. et Zucc. ZGYD is mainly used in clinics for the treatment of perimenopausal syndrome, often combined with other drugs [3, 4]. In the combined use of traditional Chinese medicine (TCM), Guishao Zuo-Gui Yin in the treatment of vulvar dystrophy with the syndrome of “Yin” deficiency of liver and kidney has good efficacy and few adverse reactions. It also could reduce the clinical symptoms and improve the immune function [5]. Therefore, ZGYD is worthy of clinical promotion and application. In the combined use of Western medicine, Zuoguiyinjiawei decoction combined with donepezil can significantly improve the cognitive level of patients with Parkinson's combined cognitive impairment. It was found that Zuoguiyinjiawei decoction plus has no serious adverse events during the study, indicating that Zuoguiyinjiawei decoction could treat Parkinson's combined cognitive dysfunction as a safe treatment.

In recent times, there have been burgeoning reports on the interaction between TCMs and the synergistic effects between TCM and western medicine [6, 7]. A rational combination of drugs has a synergistic effect, and an irrational combination of drugs may lead to ineffective treatment effects and even toxic side effects [8]. Interactions between metabolic drugs are primarily caused by the induction or inhibition of the production of metabolic enzymes, of which the cytochrome P450 enzyme dominates.

CYP450 is the primary metabolic enzyme system that participated in the biotransformation of endogenous and exogenous substances [9], including drugs *in vivo*. Changes in the activity of this enzyme can directly affect the changes in the *in vivo* kinetics of exogenous substances and cause subsequent biological effects [10]. For instance, catalpol, the key active ingredient in *Rehmannia glutinosa* Libosch., was shown to inhibit the activity of CYP3A4, CYP2E1, and CYP2C9 [11]. Another study has reported that *Lycium barbarum* polysaccharide (LBP) improved liver injury induced by di-2-ethylhexyl phthalate (DEHP) in rats and that PXR, CYP450, CYP2E1, CYP3A1, UGT1, and GST levels were reduced after LBP treatment [12]. Monosidine, an iridoid glycoside compound extracted from the Chinese herbal medicine *Cornus officinalis* Sieb. et Zucc., was found to significantly induce CYP3A, mRNA, and protein expression in rats [13]. Pachymic acid (a triterpenoid contained in *Poria cocos* (Schw.) Wolf) was found to inhibit the activity of CYP3A4, 2C9, and 2E1, suggesting a potential drug interaction between pachymic acid and drugs metabolized by these enzymes [14].

ZGYD is often used in combination with TCM and Western medicine, but the study on the interaction of ZGYD and drugs has not been reported. Therefore, in the preset study, the effects on CYP1A2, CYP2B1, CYP2C7, CYP2C11, CYP2D2, and CYP3A1 were developed from an *in vivo* perspective using a “Cocktail” probe drug method [15–18]. Simultaneously, RT-PCR and Western blot were used to study the effects of ZGYD on the regulation of six main metabolic enzymes. Our study provides help for the scientific and reasonable use of ZGYD.

## 2. Materials and Methods

### 2.1. Chemicals and Reagents

All the experimental herbs were produced by Anhui Puren Traditional Chinese Medicine Pieces Co., Ltd. (Hefei, China). Professor Nianjun Yu of Anhui University of Traditional Chinese Medicine determined that the herbs complied with the regulations. Probe drugs including phenacetin, bupropion, amodiaquine, omeprazole, dextromethorphan, and midazolam (purity > 98%) were gained from the National Institute for Food and Drug Control (Beijing, China). The internal standard (IS) glibenclamide was obtained from Yuanye Biotechnology Co., Ltd. (Shanghai, China). Both acetonitrile and methanol were chromatographic pure grade, while other reagents were of analytical grade or better.

### 2.2. Animals

12-week-old adult, healthy SD rats (200~240 g) were acquired from the Animal Laboratory Center of Anhui Medical University (Hefei, China), certificate number SCXK (wan) 2017-001. Animals were cultivated in a breeding room at constant 22 ± 2°C and 55 ± 10% relative humidity. After one-week adaptive feeding, the experiment was commenced. All animal experiments were conducted following approval from the Research Ethics Committee of Anhui University of Traditional Chinese Medicine (AHUCM-rats-2021118).

### 2.3. Preparation of ZGYD

According to the record of “Jing Yue Quanshu,” it is said to take cooked *Rehmannia glutinosa* Libosch. (9 g), *Dioscorea opposite* Thunb. (6 g), *Lycium barbarum* L. (6 g), *Glycyrrhiza uralensis* Fisch. (3 g), *Poria cocos* (Schw.) Wolf (4 g), and *Cornus officinalis* Sieb. et Zucc (5 g), according to the traditional decocting method, the medicinal herbs were soaked for 30 min and then decocted twice in water to obtain a filtrate and concentrated to 1.5 g/mL.

### 2.4. HPLC Analysis of ZGYD

High-performance liquid chromatography (HPLC) was performed to support the stability and quality of the ZGYD extract. The following chromatographic conditions were used: a Thermo Fisher Ultimate 3000 system with an Agilent 5 HC-C18 column (250 nm × 4.6 nm; 5 *μ*m), a column temperature of 30°C, a flow rate of 1 mL·min^−1^, a wavelength of 250 nm, and 0.1% phosphoric acid aqueous solution (A)-acetonitrile (B) gradient elution described as follows: 0-10 min, 95-93% (A); 10-20 min, 93-89% (A); 20-35 min, 89-85% (A); 35-55 min, 85-82% (A), 55-63 min, 82-72% (A); 63-73 min, 72-5% (A); and 73-78 min, 5-5% (A).

### 2.5. Preparation of Probe Cocktail Solution

The proper amount of six probe substrates were accurately weighed first, then a certain amount of anhydrous ethanol and Tween 80 was added, and finally, the volume was fixed to 10 mL with normal saline. All probe drug solutions were prepared ready to use and administered to experimental animals in volumes of 2.5 mL/kg.

### 2.6. Plasma Pharmacokinetic

Twenty-four SD rats were randomly divided into four groups (*n* = 6), including a control group (CG) and three pretreatment groups ZGYD high (HG +31 g/kg), ZGYD medium (MG +21.67 g/kg), and ZGYD low (LG +13.78 g/kg). Rats in CG were fed with a quantity of normal saline (10 mL/kg). After one week of adaptation, rats were orally administered the respective doses of ZGYD or normal saline intragastrically once daily in the morning for 14 days. On the last day, rats were given Cocktail probe solution through the tail vein. At 0.05, 0.083, 0.167, 0.25, 0.5, 0.75, 1, 2, 4, 6, 8, and 12 h after administration, blood was drawn from the venous plexus of the fundus ocularis, approximately 0.25 mL at each time. The blood samples were then placed into heparinized centrifuge tubes and centrifuged at 3500 rpm for 10 min to prepare plasma samples. All supernatant was taken and stored at -80°C.

### 2.7. Plasma Sample Preparation

90 *μ*L of rat blank plasma was precisely aspirated and placed in 1.5 mL centrifuge tube, and then, 10 *μ*L of 500 ng/mL internal standard solution and 10 *μ*L of mixed probe solution were, respectively, added into the tube. Finally, 1 mL ethyl acetate was added to precipitate the protein. The samples were centrifuged at 10 min for 12000 rpm [19]. Subsequently, the supernatant was transferred to UPLC-MS/MS for analysis.

### 2.8. Analytic Conditions

The following chromatographic conditions were used: an Agilent 1290 Infinity UPLC system with an Acquity BEH C_18_ column (2.1 mm × 100 mm, 1.7 *μ*m), a column temperature of 30°C, a flow rate of 0.2 mL/min, an injection volume of 5 *μ*L, and acetonitrile (A) -0.01% phosphoric acid aqueous solution (B) gradient elution described as follows: 0.01-1 min (10%→80% A), 1-1.3 min (80% A), 1.3-2 mi (80%→95% A), 2-3 min (95%→90% A), and 3-3.5 min (90%→10% A).

The mass spectra were detected by ESI positive ion model, collected by Multiple Reaction Monitoring in positive ion model. The capillary voltage was 3.6 kV, the ion source temperature was 400°C, and the desolventizing temperature was 500°C. The spray gas was nitrogen and the collision gas was argon. The mass spectral parameters for each compound are shown in Table 1.

### 2.9. Validation of “Cocktail” Method

#### 2.9.1. Specificity

The chromatograms of rats' plasma samples were measured under selected analytical conditions by injection of samples of rat blank plasma. Probe substrate standard plus internal standard solution, blank plasma plus probe substrate standard, glibenclamide standard, probe substrate, and internal standard chromatogram after injection of probe substrate reference substance into rat tail vein were interfered with by endogenous substances in rat blank plasma. And then, whether the probe drug in the plasma after administration was consistent with the addition of the mixed probe drug to rat blank plasma was observed.

#### 2.9.2. Standard Curves

Plasma samples containing six kinds of probe substrate concentrations were prepared by accurately drawing 90 *μ*L of rat blank plasma into 1.5 mL centrifuge tube and adding 10 *μ*L of mixed probe solution of different concentrations. Their concentrations were phenacetin (1000, 800, 400, 200, 50, 10, 1, 0.5, and 0.25 ng/mL), bupropion (600, 480, 300, 150, 100, 50, 25, 10, and 5 ng/mL), amodiaquine (200, 160, 80, 40, 20, 10, 5, 1, and 0.5 ng/mL), omeprazole (800, 640, 400, 200, 100, 50, 25, 10, and 5 ng/mL), dextromethorphan (400, 320, 160, 80, 40, 20, 10, 1, and 0.25 ng/mL), and midazolam (600, 480, 300, 150, 100, 50, 10, 1, and 0.25 ng/mL). The linear range was determined according to the preset method, and the standard curve and regression equation were determined.

#### 2.9.3. Quantitative Lower Limit

Plasma samples containing six probe drugs at concentrations of 0.25, 5, 0.5, 0.25, 0.25, and 0.25 ng/mL were prepared in the same procedure as above and analyzed according to the set analytical conditions. The measured peak area concentrations of the six probe drugs were recorded and the RSD was calculated.

#### 2.9.4. Precision and Accuracy

Precisely aspirate 90 *μ*L of rat blank plasma with 1.5 mL of the centrifuge tube, and 10 *μ*L of mixed probe solutions of different concentrations was configured and added into quality control samples of LLOQ, LOQ, MOQ, and HOQ. The concentrations of each probe drug were phenacetin (0.25, 0.5, 50, and 800 ng/mL), bupropion (5, 10, 25, and 480 ng/mL), amodiaquine (0.5, 1, 5, and 160 ng/mL), omeprazole (5, 10, 25, and 640 ng/mL), dextromethorphan (0.25, 0.5, 25, and 320 ng/mL), and midazolam (0.25, 0.5, 25, and 480 ng/mL). The intraday and interday precision of the measured concentrations were calculated from the peak areas of the internal standard glibenclamide and the six probe drugs according to the set analytical method. The ratio of the peak areas of the internal standard glibenclamide and the six probe drugs was brought into the standard curve to obtain the measured concentrations. The accuracy was expressed as the ratio of the measured concentrations to the indicated concentrations, and the RSD values were calculated.

#### 2.9.5. Matrix Effects

Matrix the sample group, blank rat plasma was taken and prepared into three different concentrations of plasma samples containing the probe drugs at low, medium, and high concentrations. The plasma samples were processed and assayed according to the set-up method, and the peak areas of the six probe drugs and internal standards were recorded.

For the matrix control group, the procedure was the same as that for the matrix sample group, except that rat plasma was replaced with water. The ratio of the peak areas of the matrix sample group to the peak areas of the matrix control group is calculated. It was calculated to investigate the effect of endogenous components on the six probe drugs and the internal standard.

### 2.10. RT-PCR Analysis

Total RNA extraction was performed using TRIzol reagents (Invitrogen, Carlsbad, CA, USA) in compliance with the manufacturer's instructions and the reverse transcription of RNA of acceptable purity according to the instructions of the kit [20, 21]. The results of genes expression were calculated using the comparative CT method (2^−*ΔΔ*CT^), and the primers (Zhenwo, Hefei, China) used in our study are shown in Table 2.

### 2.11. Western Blot Analysis

The *β-actin* (1 : 1000, TA-09, ZSGB-BIO, Beijing, China) was used as an internal control. The primary antibodies used were CYP2C11 (1 : 1000, bs-14178R, Bioss, Beijing, China) and CYP3A1 (1 : 1000, bs-20586R, Bioss, Beijing, China). The secondary antibodies were peroxidase-conjugated goat anti-mouse IgG (1 : 1000, #A21010, Abbkine, USA) and goat anti-rabbit IgG (1 : 1000, #A21020, Abbkine, USA).

### 2.12. Data Processing and Analysis

Noncompartmental analysis was conducted by DAS2.0 software. All values including pharmacokinetic parameters of six kinds of probe drugs were expressed as mean ± standard deviation. The mRNA expressions were performed by 2^−*ΔΔ*CT^ calculation. One-way analysis of variance (ANOVA) from SPSS software (IBM SPSS 23.0 software, IBM) was used to analyze the pharmacokinetic parameters of probe drugs in all dose groups as well as the control group of ZGY. Differences were considered to be statistically significant when *p* < 0.05.

## 3. Results

### 3.1. Analysis of ZGYD HPLC Fingerprint

**Figure 1:**
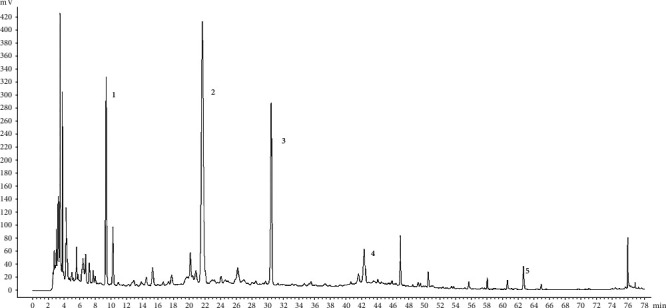
Identification of six components in ZGYD by HPLC (peak 1: 5-HMF, peak 2: morroniside, peak 3: loganin, peak 4: gentiopicroside, and peak 5: glycyrrhizic acid).

Figure 1 presents the HPLC fingerprint analysis of ZGYD. The six peaks are 5-HMF (peak 1), morroniside (peak 2), loganin (peak 3), gentiopicroside (peak 4), and glycyrrhizic acid (peak 5).

### 3.2. Validation of “Cocktail” Method

#### 3.2.1. Specificity

**Figure 2:**
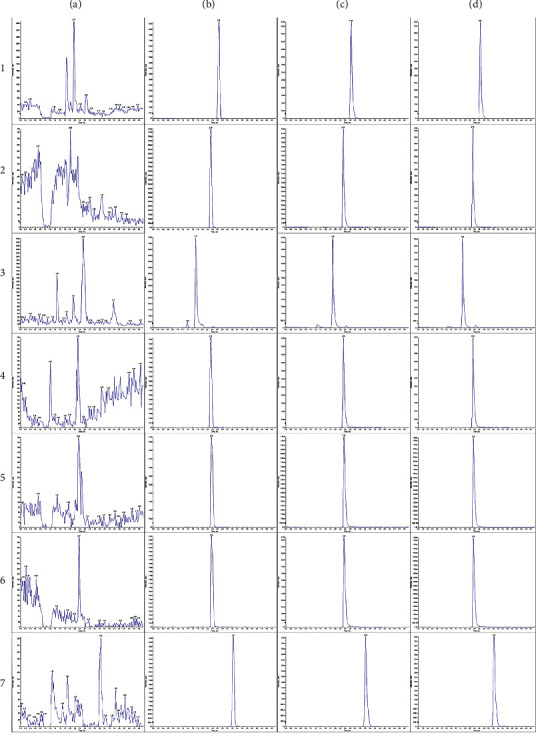
UPLC-MS/MS chromatogram of six probe drugs and the internal standard in rat plasma. (a) Blank plasma chromatogram. (b) Chromatogram of probe substrate standard plus internal standard solution. (c) Chromatogram of blank plasma plus probe substrate standard and glibenclamide standard. (d) Probe substrate and internal standard chromatogram after injection of probe substrate reference substance into rat tail vein. 1: phenacetin, 2: bupropion, 3: amodiaquine, 4: omeprazole, 5: dextromethorphan, 6: midazolam, and 7: glibenclamide.

As can be seen in Figure 2 that under the established chromatographic conditions, the impurity peaks in rat plasma had essentially no interference with the probe drugs and internal standards. And the internal standards of six probe substrates had good peak shapes, complete separation, and consistent retention behavior. The result indicated that the UPLC-MS/MS method could be used for quantitative analysis.

#### 3.2.2. Linear Ranges and Standard Curves

The linear ranges and regression equations of the six probe drugs are shown in Table 3. The linear ranges of the six probe substrates were 0.25-1000.00, 5.00-600.00, 0.50-200.00, 5.00-800.00, 0.25-400.00, and 0.25-600.00 (ng/mL), and the correction coefficients (*r*) were 0.9965, 0.9989, 0.9984, 0.9972, 0.9991, and 0.9954, respectively. The findings revealed that the drug linearity of each probe was good and it could meet the requirement of biological sample analysis.

#### 3.2.3. Quantitative Lower Limit

Table 4 and Supplementary Table 1 show that the RSD of each probe drug was less than 15% [16], which indicated that the sensitivity of the established method was high and met the requirements of biological sample analysis.

#### 3.2.4. Precision and Accuracy

Data of six kinds of probe drugs interday as well as intraday precision in plasma at LLOQ, LOQ, MOQ, and HOQ concentrations are shown in Table 4 and Supplementary Table 2. Moreover, the accuracy including LLOQ, LOQ, MOQ, and HOQ concentrations of plasma are assessed in Table 4 and Supplementary Table 3. The results reflected that each kind of RSD was all below 15% [19]. Precision and accuracy were both fine and met the criteria of the biological sample analysis.

#### 3.2.5. Matrix Effects

Table 4 and Supplementary Table 4 indicate that the matrix effects of six probes drugs and glibenclamide ranged from 85% to 115%. The results also showed that the endogenous components in the plasma samples did not affect the results of the probe substrate determination and could be used for the quantitative analysis of the substrate.

### 3.3. Pharmacokinetics Parameters

#### 3.3.1. Effects of ZGYD on the Activities of Rat CYP1A2, CYP2B1, and CYP2C7

**Figure 3:**
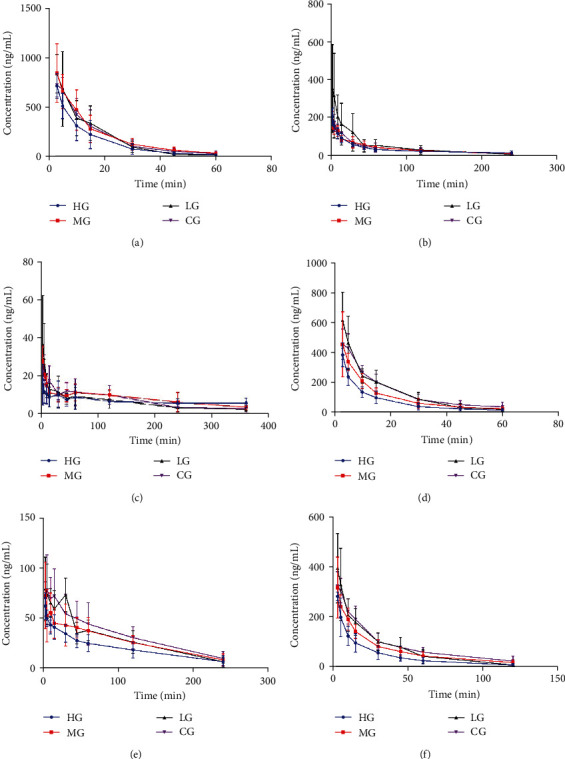
(a) The mean concentration-time curve of phenacetin in rat (ng/mL). (b) The mean concentration-time curve of bupropion in rats (ng/mL). (c) The mean concentration-time curve of amodiaquine in rats (ng/mL). (d) The mean concentration-time curve of omeprazole in rats (ng/mL). (e) The mean concentration-time curve of dextromethorphan in rats (ng/mL). (f) The mean concentration-time curve of midazolam in rats (ng/mL).

Pharmacokinetic profiles of phenacetin, bupropion, and amodiaquine were used in the study group to investigate the activity of CYP1A2, CYP2B1, and CYP2C7, respectively [18, 22, 23]. The mean drug-time curves and main pharmacokinetics parameters for the different groups are shown in Figures 3(a)–3(c) and Tables 5(a)–5(c), respectively. Compared to the control group, the main pharmacokinetic parameters of ZGYD in high-, middle-, and low-dose groups showed that no significant changes, suggesting that ZGYD does not affect CYP1A2, CYP2B1, and CYP2C7 activity *in vivo*.

#### 3.3.2. Effect of ZGYD on the Activities of Rat CYP2C11

Changes in CYP2C11 activity were depicted in the main pharmacokinetic parameters and mean plasma concentration-time curves illustrated in Figure 3(d) and Table 5(d). The result presented that in comparison with the control group, ZGYD in the HG significantly decreased AUC_(0-t)_ and AUC_(0-∞)_ approximately 1.94-fold and 1.93-fold (*p* < 0.01, *p* < 0.01), while CL was significantly increased approximately 2.0-fold (*p* < 0.05). According to the results, the CYP2C11 enzyme activity was induced, thereby accelerating metabolism and reducing plasma drug concentrations.

#### 3.3.3. Effect of ZGYD on the Activities of Rat CYP2D2

Dextromethorphan was metabolized by CYP2D2 in rats [23]. The mean drug-time curves of the HG, MG, LG, and CG after the probe solution were fitted as shown in Figure 3(e). Using DAS2.0 software, the data were fitted by statistical moment method of the noncompartment model. The pharmacokinetics parameters of the high-, medium-, and low-dose groups and control groups were obtained, as shown in Table 5(e). Compared with the control group, the AUC_(0-t)_ and AUC_(0-∞)_ decreased significantly (*p* < 0.01, *p* < 0.05), about 1.56-fold and 1.78-fold, while CL increased significantly (*p* < 0.05), about 2.0-fold, suggesting that the ZGYD in the HG could induce CYP2D2 activity *in vivo*.

#### 3.3.4. Effect of ZGYD on the Activities of Rat CYP3A1

The CYP3A1 activity was investigated by analyzing the pharmacokinetic parameters of midazolam [24].  Figure 3(f) and Table 5(f) display that compared to the CG, the AUC_(0-t)_ and AUC_(0-∞)_ of the ZGYD in the HG decreased significantly about 2.38-fold and 2.57-fold (*p* < 0.01, *p* < 0.01). The CL increased significantly about 2.68-fold (*p* < 0.05), suggesting that the ZGYD affects the drug metabolism mediated by CYP3A1.

#### 3.3.5. Effects of ZGYD on CYP2C11, CYP2D2, and CYP3A1 mRNA and Protein Expressions in Rats

**Figure 4:**
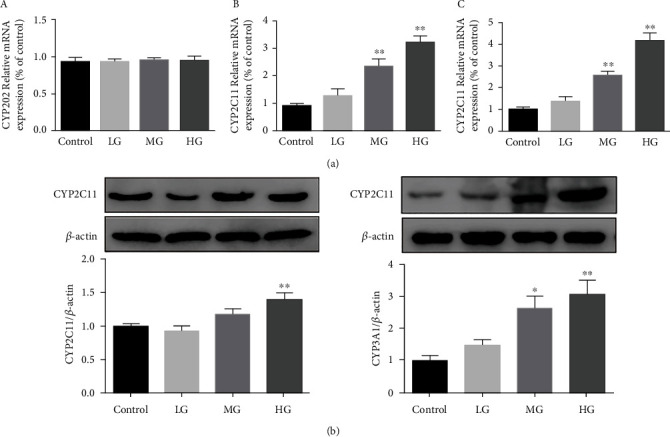
(a) Effects of ZGYD on the mRNA expression of CYP2D2 CYP2C11 and CYP3A1 in rat liver (compared to CG, ^∗∗^*p* < 0.01). A CYP2D2 relative mRNA. B CYP2C11 relative mRNA. C CYP3A1 relative mRNA. (b) Effects of ZGYD on the protein expression of CYP2C11 and CYP3A1 in rat liver (compared to CG, ^∗^*p* < 0.05 and ^∗∗^*p* < 0.01).

The results of PCR are shown in Figure 4(a) and Table 6. As compared to the CG, both the MG and HG of ZGYD could significantly upregulate the mRNA expression of CYP2C11 and CP3A1 enzymes, while each dose group had no significant effect on the mRNA expression of CYP2D2 enzymes. Similarly, the results of Western blot experiments are shown in Figure 4(b). It can be seen that the ZGYD high-dose group could significantly upregulate CYP2C11 expression (*p* < 0.01) and the medium-dose group and high-dose group could significantly upregulate CYP3A1 protein expression (*p* < 0.05, *p* < 0.01). However, each dose group does not affect CYP2D2 enzyme protein expression; these results were consistent with those obtained by RT-PCR.

## 4. Discussion

For the dosage of ZGYD, the clinical dose of ZGYD was usually 3.307 g/kg as the human drug dose [4]. Body mass coefficient was used to change this dose to the corresponding dose administered to rats. We finally determined high, medium, and low doses of 31 g/kg, 20.67 g/kg, and 13.78 g/kg for rats, respectively [4]. In addition, this study also used gavage administration in rats because of the clinical and practical use of ZGYD, which is the closest way to human dosing.

CYP450 enzymes differ markedly by species, sex, and age, and none of the animals are exactly similar to humans in terms of activity of all CYP enzymes [25]. Since rats have the similarity CYP1A2 as humans, it has been demonstrated that CYP2B1, CYP2C7, CYP2C11, CYP2D2, and CYP3A1 enzymes in rat liver microsomes correspond to CYP2B6, CYP2C8, CYP2C19, CYP2D6, and CYP3A4 enzymes in human liver microsomes, respectively [26–29]. Therefore, it is possible to study the effect of the ZGYD on CYP1A2, CYP2B1, CYP2C7, CYP2C11, CYP2D2, and CYP3A4 subtypes of enzymes in the rat liver.

Human cytochrome P450 1A2 (CYP1A2) is one of the major CYPs in the liver (~13%). Approximately one-fifth of clinically used drugs are metabolized by it [30]. Among them are certain antipyretic and analgesic medications, such as paracetamol and naproxen [31]. Compared with CYP1A2, CYP2B enzymes (<1% of total CYP) exhibit a relatively low level of catalytic preservation across mammalian organisms [32]. In SD rats, CYP2B is mainly present in the form of CYP2B1, while in humans, it is CYP2B6 that predominates [33]. The two enzymes share 97% sequence homology and very similar substrate specificity, with catalytic activity being the main difference between the two enzymes, usually higher for CYP2B1 [34]. CYP2C7 gene is associated with the CYP2C subfamily of the rats [35]. This subfamily proved primarily involved in the stereospecific metabolism of steroids and typically exhibits sex-differentiated expression. Additionally, the 2C7 type catalyzes the oxidation of retinol and retinoic acid into polar metabolites, suggesting their participation in hepatic regulation of vitamin A metabolism [36]. In this study, we found that ZGY did not affect rat CYP1A2, CYP2B1, and CYP2C7 activity *in vivo*. Based on this, ZGYD can be used in many therapeutic applications along with CYP1A2-, CYP2B1-, and CYP2C7-metabolized drugs, but individual differences should also be considered.

CYP2D2 enzyme is known to metabolize most of the usual substrates of the human CYP2D6 enzyme [29], the most commonly characterized polymorphic drug-metabolizing enzyme [37]. In this experiment, we found that the high-dose group of ZGYD can induce the activity of CYP2D2 in rats, while low-, middle-, and high-dose groups had no significant effect on CYP2D2 mRNA and protein expressions. It is clear from this conclusion that there is no need to be concerned about adverse drug interactions when ZGYD is used in combination with drugs metabolized by CYP2D2.

CYP2C11 is not only a rat orthologue of human CYP2C19 but also the most abundant isoform of CYPs in male rats [38]. It has participated in the biotransformation of endogenous substances such as epoxidation of arachidonic acid, hydroxylation of testosterone, androgen diketone, and vitamin D [39, 40], and about 10% to 20% of clinical use of medication, including phenytoin [41], tolbutamide [42], and warfarin with narrow therapeutic indices, are metabolized by this enzyme. CYP3A4 is one of the most abundant hepatic CYP450 isoforms involved in the biotransformation of various drugs and environmental chemicals accounting for approximately 30% of all human hepatic CYP450s [43]. Besides, the rat liver CYP3A subfamily has been extensively studied in various nonclinical drug metabolism studies, and the experimental results are often used to assess changes in drug metabolism in human clinical situations [44]. In terms of metabolism, the most relevant isoform of CYP3A1 in the rat is the orthologue of human CYP3A4, with 73% amino acid homology to human CYP3A4 [45]. According to our experimental results, the ZGYD high-dose group induced effects on CYP2C11 and CYP3A4, and this effect may be achieved by modulating the gene expression of both and thus altering protein function. But whether this consistency is species-specific requires further experimentation to verify.

Taken together, our results indicated that high doses of ZGYD were found to have an inductive effect on CYP2C11 and CYP3A1. However, no significant change was observed in CYP1A2, CYP2B1, CYP2C7, and CYP2D2 activities. It suggested that we should not only pay attention to the combination of ZGYD with drugs related to CYP2C11 and CYP3A1 metabolism (CYP2C19 and CYP3A4 in humans), but herbal drug interactions (HDIs), individual differences, and doses should also be analyzed when using ZGYD.

## 5. Conclusion

In summary, this paper evaluated the activity and mRNA expression of ZGYD on six different CYP450 enzymes in rats using a cocktail probe method based on UPLC-MS/MS, PCR, and Western blot techniques. The results indicated that ZGYD may have inducing effects on CYP2C11 and CYP3A1 (CYP2C19 and CYP3A4 in humans) in rats. However, no significant change in CYP1A2, CYP2B1, CYP2C7, and CYP2D2 activities was observed. It also suggested that drug interactions need to be noted when ZGYD is combined with drugs metabolized via CYP2C19 and CYP3A4. However, further experiments and clinical trials are needed to support our experimental conclusions.

## Figures and Tables

**Table 1:** Mass spectrum parameters of probe drugs and glibenclamide.

Compounds	Parent ion (m/z)	Product ion (m/z)	Collision energy (V)
Phenacetin	180.1	109.9	35
Bupropion	240.0	183.9	16
Amodiaquine	356.3	283.0	25
Omeprazole	346.1	197.9	14
Dextromethorphan	272.1	171.1	53
Midazolam	326.1	290.6	35
Glibenclamide	493.9	169.1	46

**Table 2:** Oligonucleotide primers used for real-time PCR.

CYPs	Forward primers	Reverse primers
CYP2C11	GGAGGAACTGAGGAAGAGCA	AATGGAGCATATCACATTGCAG
CYP2D2	GAAGGAGAGCTTTGGAGAGGA	AGAATTGGGATTGCGTTCAG
CYP3A1	TGCCAATCACGGACACAGA	ATCTCTTCCACTCCTCATCCTTAG
*β*-Actin	GCCCAGAGCAAGACAGGTAT	GGCCATCTCCTGCTCGAAGT

**Table 3:** Linear regression equation of each probe drug in rat plasma (*n* = 3).

Compounds	Liner range (ng/mL)	Regression equation	Correlation coefficient (*r*)
Phenacetin	0.25~1000.00	*y* = 0.0864*x* + 0.2944	0.9965
Bupropion	5.00~600.00	*y* = 0.0686*x* + 0.2092	0.9989
Amodiaquine	0.50~200.00	*y* = 0.1103*x* + 0.0669	0.9984
Omeprazole	5.00~800.00	*y* = 0.0931*x* + 0.1020	0.9972
Dextromethorphan	0.25~400.00	*y* = 0.0579*x* + 0.3466	0.9991
Midazolam	0.25~600.00	*y* = 0.047*x* + 0.4590	0.9954

**Table 4:** The method validation of six probe drugs in rat plasma sample.

Compounds	RSD (%) of probe drugs						
	Lower limit	Interday precision	Intraday precision	Accuracy	Matrix effect		
					LQC	MQC	HQC
Phenacetin	4.92	5.73	10.11	5.64	6.73	2.40	1.17
		4.83	8.83	9.26			
		2.22	4.17	2.50			
		3.45	2.99	0.70			

Bupropion	5.60	2.62	4.68	4.42	4.33	1.91	4.50
		5.75	1.11	3.22			
		4.53	6.32	3.06			
		3.00	2.32	2.05			

Amodiaquine	8.88	11.80	9.45	2.14	4.73	1.94	2.85
		4.97	5.23	5.10			
		5.44	8.59	4.49			
		4.38	2.63	1.51			

Omeprazole	5.60	6.05	8.11	5.00	3.02	0.95	2.46
		8.36	1.62	3.57			
		4.20	1.73	2.74			
		2.49	5.97	1.40			

Dextromethorphan	4.43	14.43	7.14	13.61	4.15	7.41	2.31
		12.51	9.78	7.46			
		3.01	7.13	5.30			
		2.74	2.55	1.30			

Midazolam	11.49	7.83	10.36	9.31	4.88	3.81	3.64
		9.83	11.47	10.51			
		2.53	1.15	1.79			
		4.01	3.60	1.08			

**Table:** (a) Main pharmacokinetic parameters of phenacetin in rat plasma ($\bar{x}\pm s$, *n* = 6)

Parameter	CG	LG	MG	HG
AUC_(0-t)_ (ng/mL·min)	13.46 ± 3.67	13.47 ± 4.38	14.48 ± 4.70	10.28 ± 3.62
AUC_(0-∞)_ (ng/mL·min)	14.33 ± 3.70	13.85 ± 4.35	15.42 ± 4.71	10.85 ± 3.52
MRT_(0-∞)_ (min)	19.36 ± 9.15	14.20 ± 3.32	18.97 ± 3.04	17.60 ± 11.36
T_1/2_ (min)	16.65 ± 8.31	11.70 ± 4.05	16.56 ± 4.01	15.60 ± 11.37
CL (L/min/kg)	0.074 ± 0.018	0.079 ± 0.027	0.072 ± 0.028	0.099 ± 0.026

**Table:** (b) Main pharmacokinetic parameters of bupropion in rat plasma ($\bar{x}\pm s$, *n* = 6)

Parameter	CG	LG	MG	HG
AUC_(0-t)_ (ng/mL·min)	8.70 ± 1.98	13.57 ± 8.070	8.27 ± 1.73	8.03 ± 1.71
AUC_(0-∞)_ (ng/mL·min)	9.90 ± 4.06	14.17 ± 8.05	9.20 ± 1.81	11.09 ± 6.61
MRT_(0-∞)_ (min)	78.01 ± 17.68	59.68 ± 29.22	95.38 ± 7.04	97.88 ± 38.96
T_1/2_ (min)	56.29 ± 18.80	55.77 ± 31.93	70.82 ± 12.73	66.24 ± 17.78
CL (L/min/kg)	0.11 ± 0.03	0.09 ± 0.053	0.11 ± 0.02	0.11 ± 0.05

**Table:** (c) Main pharmacokinetic parameters of amodiaquine in rat plasma ($\bar{x}\pm s$, *n* = 6)

Parameter	CG	LG	MG	HG
AUC_(0-t)_ (ng/mL·min)	2.45 ± 0.42	2.25 ± 1.18	2.72 ± 1.06	2.41 ± 0.62
AUC_(0-∞)_ (ng/mL·min)	3.42 ± 1.75	2.52 ± 1.44	3.68 ± 1.65	5.80 ± 4.17
MRT_(0-∞)_ (min)	214.42 ± 45.36	177.21 ± 37.72	257.51 ± 140.72	548.37 ± 476.07
T_1/2_ (min)	119.08 ± 43.61	105.13 ± 38.34	176.49 ± 109.09	238.03 ± 136.05
CL (L/min/kg)	0.34 ± 0.13	0.52 ± 0.29	0.32 ± 0.15	0.25 ± 0.16

**Table:** (d) Main pharmacokinetic parameters of omeprazole in rat plasma ($\bar{x}\pm s$, *n* = 6)

Parameter	CG	LG	MG	HG
AUC_(0-t)_ (ng/mL·min)	9.47 ± 2.36	9.77 ± 1.71	7.35 ± 1.19	4.86 ± 1.28^∗∗^
AUC_(0-∞)_ (ng/mL·min)	10.33 ± 3.02	10.64 ± 1.85	8.13 ± 1.78	5.34 ± 1.50^∗∗^
MRT_(0-∞)_ (min)	22.71 ± 8.97	15.76 ± 4.08	21.65 ± 9.20	20.16 ± 10.75
T_1/2_ (min)	16.64 ± 6.43	12.22 ± 3.23	18.99 ± 9.95	16.53 ± 8.82
CL (L/min/kg)	0.10 ± 0.02	0.10 ± 0.01	0.12 ± 0.02	0.20 ± 0.05^∗^

Compared to CG, ^∗^*p* < 0.05 and ^∗∗^*p* < 0.01.

**Table:** (e) Main pharmacokinetic parameters of dextromethorphan in rat plasma ($\bar{x}\pm s$, *n* = 6)

Parameter	CG	LG	MG	HG
AUC_(0-t)_ (ng/mL·min)	7.59 ± 1.80	7.22 ± 2.34	5.69 ± 1.41	4.85 ± 1.41^∗∗^
AUC_(0-∞)_ (ng/mL·min)	10.32 ± 5.01	8.18 ± 2.93	8.18 ± 1.93	5.79 ± 2.03^∗^
MRT_(0-∞)_ (min)	135.44 ± 43.22	94.44 ± 33.87	157.32 ± 60.56	126.69 ± 67.94
T_1/2_ (min)	95.84 ± 36.02	68.73 ± 32.51	119.85 ± 45.12	81.41 ± 45.33
CL (L/min/kg)	0.11 ± 0.04	0.14 ± 0.07	0.12 ± 0.03	0.21 ± 0.14^∗^

Compared to CG, ^∗^*p* < 0.05 and ^∗∗^*p* < 0.01.

**Table:** (f) Main pharmacokinetic parameters of midazolam in rat plasma ($\bar{x}\pm s$, *n* = 6)

Parameter	CG	LG	MG	HG
AUC_(0-t)_ (ng/mL·min)	10.87 ± 2.49	9.34 ± 3.15	8.74 ± 1.79	4.55 ± 1.53^∗∗^
AUC_(0-∞)_ (ng/mL·min)	13.01 ± 4.87	10.35 ± 3.36	9.65 ± 2.46	5.06 ± 1.44^∗∗^
MRT_(0-∞)_ (min)	57.50 ± 31.29	44.47 ± 47.53	47.128 ± 11.66	28.97 ± 11.39
T_1/2_ (min)	44.89 ± 27.54	42.36 ± 58.68	35.601 ± 14.01	23.96 ± 16.30
CL (L/min/kg)	0.08 ± 0.02	0.11 ± 0.05	0.11 ± 0.02	0.21 ± 0.06^∗∗^

Compared to CG, ^∗^*p* < 0.05 and ^∗∗^*p* < 0.01.

**Table 6:** Effects of ZGYD on the expression of CYP2C11, CYP2D2, and CYP3A1 in rat liver ($\overset{¯}{x}\pm s$, *n* = 3).

Genes	CG	ZGYD-L	ZGYD-M	ZGYD-H
*CYP2C11*	0.926 ± 0.066	1.288 ± 0.244	2.354 ± 0.273^∗∗^	3.243 ± 0.220^∗∗^
*CYP2D2*	0.927 ± 0.061	0.939 ± 0.032	0.956 ± 0.027	0.954 ± 0.055
*CYP3A1*	1.011 ± 0.095	1.406 ± 0.182	2.597 ± 0.187^∗∗^	4.211 ± 0.340^∗∗^

Compared to CG, ^∗∗^*p* < 0.01.

## Data Availability

The data used to support the findings of this study are available from the corresponding author upon request.

## Abbreviations

ZGYD: Zuo-Gui Yin Decoction
CYP450: Cytochrome P450 enzyme
TCM: Traditional Chinese medicine
UPLC-MS/MS: Ultraperformance liquid chromatography/tandem mass spectrometry
HPLC: High-performance liquid chromatography
RT-PCR: Real-time polymerase chain reaction
CG: Control group
HG: High group
MG: Medium group
LG: Low group
AUC: Area under curve
T1/2: Half-life time
CL: Clearance
LLOQ: Low limit of qualification
LQC, MQC, HQC: Low, medium, and high quantification concentration
RSD: Relative standard deviation
HDIs: Herb-drug interactions.

## Supplementary Materials

Supplementary Materials Supplementary Material is available on the publisher's website along with the published article. The results of the validation of “cocktail” method are provided in Supplementary material.
