# Supplementary material for: Evaluation of Zuo-Gui Yin Decoction Effects on Six CYP450 Enzymes in Rats Using a Cocktail Method by UPLC-MS/MS
Source: Biomed Res Int. 2022 Aug 26;2022:4293062. doi: 10.1155/2022/4293062 (PMC9439930; doi:10.1155/2022/4293062)
Supplement: Supplementary Materials — Supplementary Material is available on the publisher's website along with the published article. The results of the validation of “cocktail” method are provided in Supplementary material. [file 4293062.f1.docx]

**Supplementary Table 1.** The lower limit of six probe drug in rat plasma sample(n=5)

| Compounds | Nominal concentration(ng/mL) | Mean ±SD | RSD (%) |
| --- | --- | --- | --- |
| Phenacetin | 0.25 | 0.24±0.01 | 4.92 |
| Bupropion | 5.00 | 4.98±0.27 | 5.60 |
| Amodiaquine | 0.50 | 0.51±0.04 | 8.88 |
| Omeprazole | 5.00 | 4.98±0.27 | 5.60 |
| Dextromethorphan | 0.25 | 0.25±0.01 | 4.43 |
| Midazolam | 0.25 | 0.25±0.02 | 11.49 |

**Supplementary Table 2.** Interday and intraday precision of six probe drugs in rat plasma

| Compounds | Add (ng/mL) | Interday precision | | Intraday precision | |
| --- | --- | --- | --- | --- | --- |
|  |  | Mean±SD(ng/mL) | RSD (%) | Mean±SD(ng/mL) | RSD (%) |
| Phenacetin | 0.25  0.50  50.00  800.00 | 0.25±0.01  0.51±0.02  50.81±1.13  808.10±27.94 | 5.73  4.83  2.22  3.45 | 0.25±0.02  0.50±0.04  51.03±2.13  801.13±23.99 | 10.11  8.83  4.17  2.99 |
| Bupropion | 5.00  10.00  25.00  480.00 | 5.02±0.13  10.82±0.62  25.19±1.14  480.22±14.40 | 2.62  5.75  4.53  3.00 | 4.96±0.23  10.08±0.11  25.91±1.64  482.11±11.21 | 4.68  1.11  6.32  2.32 |
| Amodiaquine | 0.50  1.00  5.00  160.00 | 0.49±0.05  1.01±0.50  5.19±0.28  157.22±6.89 | 11.80  4.97  5.44  4.38 | 0.49±0.04  1.13±0.05  4.98±0.42  152.96±4.02 | 9.45  5.23  8.59  2.63 |
| Omeprazole | 5.00  10.00  25.00  640.00 | 5.08±0.30  10.03±0.83  25.83±1.08  641.60±16.01 | 6.05  8.36  4.20  2.49 | 4.95±0.40  10.11±0.16  26.25±0.45  639.86±38.21 | 8.11  1.62  1.73  5.97 |
| Dextromethorphan | 0.25  0.50  25.00  320.00 | 0.24±0.03  0.52±0.06  24.86±0.75  320.16±8.78 | 14.43  12.51  3.01  2.74 | 0.26±0.01  0.52±0.05  25.40±1.81  31.9.99±8.71 | 7.14  9.78  7.13  2.55 |
| Midazolam | 0.25  0.50  25.00  480.00 | 0.25±0.01  0.50±0.04  24.62±0.62  485.56±19.49 | 7.83  9.83  2.53  4.01 | 0.25±0.02  0.50±0.05  24.50±0.28  483.92±17.45 | 10.36  11.47  1.15  3.60 |

**Supplementary Table 3.** The accuracy of six probe drugs in rat plasma (n=5)

| Compounds | Nominal concentration (ng/mL) | Accuracy，mean±SD (ng/mL) | RSD (%) |
| --- | --- | --- | --- |
| Phenacetin | 0.25  0.50  50.00  800.00 | 103.76±5.86  103.44±9.58  99.76±2.49  101.76±0.71 | 5.64  9.26  2.50  0.70 |
| Bupropion | 5.00  10.00  25.00  480.00 | 100.64±4.45  98.69±3.18  103.90±3.18  104.60±2.14 | 4.42  3.22  3.06  2.05 |
| Amodiaquine | 0.50  1.00  5.00  160.00 | 101.56±2.17  105.66±5.39  99.89±4.48  99.56±1.50 | 2.14  5.10  4.49  1.51 |
| Omeprazole | 5.00  10.00  25.00  640.00 | 102.76±5.14  98.11±3.50  104.88±2.87  102.28±1.44 | 5.00  3.57  2.74  1.40 |
| Dextromethorphan | 0.25  0.50  25.00  32.00 | 102.55±13.95  100.81±7.52  97.29±5.15  103.80±1.34 | 13.61  7.46  5.30  1.30 |
| Midazolam | 0.25 | 101.50±9.45  100.71±10.58  103.92±1.86  101.15±1.09 | 9.31  10.51  1.79  1.08 |
|  | 0.50 |  |  |
|  | 25.00 |  |  |
|  | 480.00 |  |  |

**Supplementary Table 4.** Matrix effect of six probe substrates and internal standard in plasma(n=6)

| Nominal concentration(ng/mL) | Substrate | M4atrix effect (%) | RSD (%) |
| --- | --- | --- | --- |
| LQC | Phenacetin  Bupropion  Dextromethorphan  Midazolam  Omeprazole  Amodiaquine  Glibenclamide | 105.72±7.12  97.71±4.32  95.26±4.50  95.11±2.87  95.18±3.95  97.08±4.74  102.34±3.39 | 6.73  4.33  4.73  3.02  4.15  4.88  3.31 |
| MQC | Phenacetin  Bupropion  Dextromethorphan  Midazolam  Omeprazole  Amodiaquine  Glibenclamide | 99.63±2.39  98.95±1.89  98.37±1.91  100.78±0.96  97.03±7.19  101.04±3.85  103.10±3.27 | 2.40  1.91  1.94  0.95  7.41  3.81  3.18 |
| HQC | Phenacetin  Bupropion  Dextromethorphan  Midazolam  Omeprazole  Amodiaquine  Glibenclamide | 99.65±1.17  99.91±4.50  100.10±2.85  98.20±2.42  106.90±2.47  100.48±3.65  96.07±2.74 | 1.17  4.50  2.85  2.46  2.31  3.64  2.85 |
